# Metformin or insulin: logical treatment in women with gestational diabetes in the Middle East, our experience

**DOI:** 10.1186/s13104-018-3540-1

**Published:** 2018-07-03

**Authors:** Sindu J. Christian, Vincent Boama, Hiba Satti, Joohi Ramawat, Tarik A. Elhadd, Khaled Ashawesh, Khaled Dukhan, Stephen Beer

**Affiliations:** 1 Department of Obstetrics and Gynecology, Al Wakra Hospital, Hamad Medical Corporation, PO Box 82228, Doha, Qatar; 2 Department of Obstetrics and Gynecology, Sidra Medical and Research Center, PO box 26999, Doha, Qatar; 3 Department of Medicine, Endocrine Section, National Diabetes Center, Al Wakra Hospital, Qatar Metabolic Institute, Doha, Qatar

**Keywords:** Gestational diabetes, Pharmacological intervention, Perinatal outcome

## Abstract

**Objective:**

The debate still continues about the preferred modality of treatment of gestational diabetes requiring pharmacological treatment. Insulin was previously considered as the gold standard, but the National Institute of Health and Care Excellence now recommend metformin as the first line drug of choice. The pharmacological management of gestational diabetes mellitus in the Middle East with its high risk population has not been widely published. We aim to evaluate the safety and efficacy of using metformin in comparison to insulin, in our group of patients, and to study key associated morbidities.

**Results:**

A total of 291 women registered in the clinic during the study period. One hundred and twenty-one (121) were women with gestational diabetes Mellitus requiring medical therapy. Among them, 107 delivered at term. Ninety (84%) women received metformin. Additional insulin was required in 32% of these patients. There was a significant difference in the birth weight of babies in the metformin with insulin group of 207 g (p value 0.04) in favour of metformin. There was no significant difference in maternal or neonatal morbidities between the groups. Metformin was thus found to be a safe, practical and cost effective medication to be offered to our population.

**Electronic supplementary material:**

The online version of this article (10.1186/s13104-018-3540-1) contains supplementary material, which is available to authorized users.

## Introduction

Gestational diabetes mellitus (GDM) is defined as any degree of glucose intolerance which develops in the 2nd or 3rd trimester of pregnancy; if the patient has no previous history [1]. This complicates around 1–14% of all pregnancies [2], the prevalence varying depending on race and ethnicity and the screening method used [3, 4]. With obesity reaching epidemic proportions, there is a proportional rise in the incidence of gestational diabetes [3, 5, 6].

GDM is associated with short and long term risk to both the mother and the foetus. There is increased risk of preeclampsia, caesarean section rate and an increased risk of development of type II diabetes mellitus in later life, for the mother [7, 8]. The foetus can be affected by macrosomia, has a higher risk of congenital defects, still birth at term, shoulder dystocia at birth and hypoglycaemia postnatally [9, 10]. There is also increased risk of long term effect on the health of the child including obesity and the metabolic syndrome [11].

The initial treatment of GDM is lifestyle modification with dietary therapy and exercise [12]. Patients not responding to the above require pharmacological therapy. The aim is to reduce the hyperglycaemia in the mother, hence significantly reducing perinatal morbidity and improving the quality of life of the woman [13, 14].

The gold standard of management of GDM is the administration of insulin [13, 15]. In spite of the development of newer and safer forms of insulin, its use is associated with increased maternal weight or hypoglycaemia. Controlling blood sugar with insulin requires intensive monitoring by patient with frequent adjustment of dosage and maintenance of the cold chain. The increased cost of treatment and training and the need for regular insulin injections with associated complications, makes it an unattractive option for some women.

Metformin is a long established drug for the management of diabetes mellitus. It increases insulin sensitivity by activating AMP kinase and reduces hepatic gluconeogenesis [16]. It is not associated with weight gain and hypoglycaemia [17]. In spite of a 10–16% risk of maternal to foetal transfer of drug [18], newer studies have not shown any adverse effect on the foetus [19]. In its latest guidelines, National Institute of Health and Care Excellence (NICE) recommends the use of metformin as a first line drug in the absence of any contraindication [20]. The ease of use and low cost makes it an potential alternative in all parts of the world regardless of ethnic background.

## Main text

### Methods

This was a prospective service evaluation of the work done at the joint obstetric diabetic clinic (JODC) at the Al Wakra hospital, Qatar. All pregnant women are routinely booked at the primary health centre. After 24 weeks, universal screening for GDM is performed using the World Health Organisation (WHO) 75 g oral glucose tolerance test (OGTT) [21] and diagnostic criteria are based on the International Association of the Diabetes and Pregnancy Study Groups (IADPSG) Threshold [22]. Patients with FBS ≥ 7.0 mmol/l (126 mg/dl), HbA1c ≥ 6.5%, Random blood glucose ≥ 11.1 mmol/l (200 mg/dl) were diagnosed as overt diabetes and hence excluded from the study. Women diagnosed with GDM, are started on diet and exercise and advised home monitoring of blood sugars. If the blood sugars are not controlled within 2 weeks, they are referred to the JODC for initiation of therapy.

We evaluated our practice from 01/07/2015 to 31/04/2016. A total of 291 patients were registered in the clinic during this period, of which 121 were GDM requiring treatment. There were 72 patients of type I or type 2 diabetes and 98 patients were with other endocrine issues. The 107 patients of GDM who delivered at term in this period were included in our evaluation.

#### Data extraction

The antepartum, intrapartum and postpartum details of the patients were taken from their electronic medical records. Maternal data included demographics, gestational age at booking, baseline Glycated haemoglobin (HbA1c), and time of initiation or change of therapy, other antenatal complications and type of delivery. Neonatal data included birthweight and any immediate neonatal complications.

#### Analysis

All data were entered into a Microsoft excel sheet. p value of outcomes were calculated using the Student’s T test. Statistical significance was awarded if p value < 0.05.

### Results

During the period of our study, 219 patients were registered in the JODC, of which 121 patients were diagnosed cases of GDM, not controlled on diet. These patients were initiated on medical treatment and 107 of them delivered at term, and were included in our review.

The age and population demographics of these patients are compared in Table 1.

**Table 1:** Baseline demographics of patients in the metformin and insulin group

	Metformin (58)	Met+ Ins (32)	Insulin (17)
Average age in years (range)	32 (22–42)	34 (25–45)	34 (20–46)
20–40	56 (96.6%)	28 (87.5%)	14 (82.4%)
41+	2 (3.4%)	4 (12.5%)	3 (17.6%)
Average parity (range)	2 (0–9)	2 (0–5)	2 (0–6)
Nulliparity	13 (22.4%)	2 (6.3%)	5 (29.4%)
1–4	42 (72.4%)	29 (90.6%)	11 (64.7%)
5+	3 (5.2%)	1 (3.1%)	1 (5.9%)
Ethnicity			
Middle Eastern	3 (5.2%)	6 (18.7%)	7 (41.2%)
Rest of Asia	45 (77.6%)	19 (59.4%)	6 (35.3%)
Africa	10 (17.2%)	7 (21.9%)	4 (23.5%)
Average BMI in kg/m^2^ (range)	30 (23–41)	32 (23–52)	35 (23–53)
18.5–24.9	4 (6.9%)	2 (6.3%)	1 (5.9%)
25–29.9	22 (37.9%)	12 (37.5%)	2 (11.7%)
30–34.9	26 (44.8%)	7 (21.9%)	6 (35.3%)
> 35	6 (10.4%)	11 (34.3%)	8 (47.1%)

We also compared their parity and booking body mass index (BMI). Less than 1% (7/107) of the subjects had normal BMI. Twenty-three percent (23%) of the total number of patients were morbidly obese with a BMI more than 35; with 47% of patients in the insulin only group being morbidly obese. Two of our patients had BMI above 50.

There was a wide variation in the gestational age of booking at the clinic and this affected the initiation of treatment for these patients. The average HbA1c at booking for these patients were similar. This is represented in Table 2.

**Table 2:** Gestational age for booking and treatment initiation

	Metformin (58)	Met+ Ins (32)	Insulin (17)
Mean gestational age at booking in weeks	31 (14–38)	28 (15–35)	29 (18–35)
Average HbA1c at booking	5.69 (51/58)	5.78 (31/32)	5.8 (16/17)
Mean gestation in weeks for treatment initiation	30.6	27.8 (metformin)/30.7 (insulin)	30.2

A total of ninety patients were on insulin. Eighty-seven patients were started initially on metformin of which, 29 patients required additional insulin. One patient was initiated on insulin and metformin together and 2 patients were given additional metformin after starting insulin. One patient had to discontinue metformin due to excessive GI symptoms and was switched to insulin.

The preferred insulin regimen in our institution is a basal bolus of intermediate acting insulin (detemir) with or without premeal short acting insulin (aspart). Table 3 looks at the distribution of patients according to treatment modality.

**Table 3:** Distribution according to insulin

	Yes	No
Met+ Ins (32)		
Intermediate acting	27	5
Short acting	18	14
Mixtard	4	28
Insulin (17)		
Intermediate acting	13	4
Short acting	17	0
Mixtard	2	0

In the insulin only group, 4 (23.5%) patients achieved euglycaemia with only premeal short acting insulin. Thirteen patients (76.5%) were on intermediate acting with premeal short acting. Two of these patients had been initially initiated on combined insulin preparations, but this was discontinued as it failed to achieve adequate glycemic control.

In the cohort of insulin plus metformin, 27 (84.4%) patients were initiated on intermediate acting insulin. 15 patients’ required additional short acting insulin and in one lady, it was discontinued and combined insulin started. Only 3 patients were managed with premeal short acting insulin alone. In the 4 (12.5%) patients on combined insulin, 1 was discontinued in preference for basal bolus regimen.

Additional file 1: Table S1 compares the antenatal complications and mode of delivery and any associated complications of the patient’s in the three groups. The average gestational age of delivery of the patients in all the groups is comparable.

Additional file 1: Table S2 compares the weight of the babies. The baby’s in the insulin group had the highest average birth weight. There is a statistically significant difference in the weight between the baby’s in the metformin group and insulin group with the P value of 0.04.

The neonatal complications are compared in Additional file 1: Table S3. A total of 16 babies were admitted into NICU of which 9 were from the metformin group, 5 from the insulin plus metformin group and 2 from the insulin group. Significant hypoglycaemia was diagnosed in 2 babies in the metformin group and 2 babies in metformin plus insulin group. There were no neonatal deaths.

### Discussion

There is a rising epidemic of obesity and proportionally the incidence of gestational diabetes is increasing [6]. Forty percent of the population in Qatar are considered obese [23]. With the high incidence of gestational diabetes, it is important that an easier and more accessible treatment option is available to patients, to prevent delay in treatment and to improve compliance.

In the three group of patients that we studied, the baseline characteristics were similar in relation to age, parity and average booking BMI. Only 6% of the cohort had normal BMI. The patient’s in the insulin group had a much higher BMI than the metformin group. This may have an additional effect on the weight of the neonate’s in the insulin group [24].

The need for additional insulin for patients on metformin, range from 20.9% by Tertti [25] to as high as 46% in the study by Rowan [26]. In our study, 32% of patients on metformin required insulin therapy in addition.

There was no significant difference in the adverse maternal or perinatal outcomes in all three groups. There was a lower rate of preeclampsia in the metformin group, which may be because metformin has complex properties on endothelial functions and reactive oxygen species production [27].

The mean gestational age for delivery was similar in all 3 groups. The number of instrumental deliveries and caesarean sections were also similar as compared to studies by Tertti [25] and Juan Gui [27].

There was however a statistically significant difference in the birth weight between the metformin only group compared with patients requiring additional insulin with metformin. The birth weight in both groups of insulin was similar. The increased weight seen in the insulin group may be a direct effect of insulin as described by Arshad [28]. Tertti [25], Juan Gui [27] and Balani [29] also report lower birth weight in the metformin group.

The incidence of macrosomia was 12% in the group requiring metformin only and 15% in the group with metformin plus insulin and only 5% in patients on insulin only. This was a surprising finding, in contrast to the decreased incidence of macrosomia with metformin in studies by Balani [29] and Niromanesh [30]. This finding is likely due to the small number of patients in our study.

A recently published study by Nachum [31] compared the use of metformin or glyburide and in case of failure of either or both, the use of insulin. The failure rate with metformin was 29% similar to our study. The difference between this study and ours was that this study compared two oral hypoglycaemic agents.

There was no statistically significant difference in the neonatal morbidity of the 3 groups except the birth weight.

### Conclusion

40 years after the start of use of metformin in pregnancy, the debate continues on its safety and use, with guidelines differing between different countries [32]. The ease of administration, the low side effect profile and the fact that it does not require a cold chain, makes it an attractive first line therapy for the high risk population we deal with in the Middle East. This will also prevent delay in the initiation of treatment and hence reduce morbidities due to delay in receiving specialized care. A multicentre study in countries in the Gulf Corporation Council, with a higher number of patients, would be useful in addressing some of the unexpected findings in our study.

## Limitations

The main limitation of our study was the limited number of patients and the short duration of the study.

## Additional file

**Additional file 1: Table S1.** Antenatal and Postnatal details: compares the antenatal complications and mode of delivery and complications. **Table S2.** Neonatal details; compares the weight of the babies. **Table S3.** Neonatal morbidity: The neonatal complications are compared.

## Abbreviations

NICE: National Institute of Health and Care Excellence (NICE)
JODC: joint obstetric diabetic clinic
GDM: gestational diabetes mellitus
NICU: neonatal intensive care unit
WHO: World health organization
OGTT: oral glucose tolerance test
IADPSG: the International Association of the Diabetes and Pregnancy Study Groups
HbA1c: glycated haemoglobin
BMI: body mass index
