# Supplementary material for: Metformin or insulin: logical treatment in women with gestational diabetes in the Middle East, our experience
Source: BMC Res Notes. 2018 Jul 3;11:426. doi: 10.1186/s13104-018-3540-1 (PMC6029353; doi:10.1186/s13104-018-3540-1)
Supplement: Supplementary file 1 — Additional file 1: Table S1. Antenatal and Postnatal details: compares the antenatal complications and mode of delivery and complications. Table S2. Neonatal details; compares the weight of the babies. Table S3. Neonatal morbidity: The neonatal complications are compared. [file 13104_2018_3540_MOESM1_ESM.docx]

**Table S1: Antenatal and Postnatal details**

|  | **Metformin (58)** | **Met+ Ins (32)** | **Insulin (17)** |
| --- | --- | --- | --- |
| **Chronic hypertension** | 2 | 1 | 1 |
| **Hypothyroidism** | 10 | 4 | 1 |
| **Precclampsia** | 2 | 1 | 2 |
| **Elective LSCS** | 11 | 16 | 4 |
| **Emergency LSCS** | 13 | 7 | 5 |
| **Instrumental delivery** | 3 | 2 | 1 |
| **Vaginal Delivery** | 31 | 7 | 7 |
| **Gestational age of delivery in weeks** | **38.5** (37 – 41) | **38.3** (37+1 – 40) | **38.4** (37 – 39+3) |

**Table S2: Neonatal details**

|  | **Metformin (58)** | **Met+ Ins (32)** | **Insulin (17)** |
| --- | --- | --- | --- |
| **Avg Birth Weight in kg** | **3287.78** | **3409.24** | **3494.56** |
| **NICU admission** | **9 (15.51%)** | **5(15.62%)** | **2(11.76%)** |
| **Macrosomia** | **7** | **5** | **1** |
|  | **Metformin**  **Vs Met+ insulin** | **Metfomin vs insulin** | **Met+ insulin vs Insulin** |
| **P value of weight** | **0.04** | **0.19** | **0.29** |

**Table S3: Neonatal morbidity**

|  | **Hypoglycaemia** | **RDS** | **Jaundice** | **HIE** | **Sepsis** | **Social** | **Anomaly** |
| --- | --- | --- | --- | --- | --- | --- | --- |
| **Metformin** | 2 | 3 | 1 | 1 | 1 | 0 | 1 |
| **Metformin+ Insulin** | 1 | 2 | 0 | 0 | 0 | 2 | 0 |
| **Insulin** | 0 | 1 | 0 | 0 | 0 | 1 | 0 |
